# Normalization of EEG activity among previously institutionalized children placed into foster care: A 12-year follow-up of the Bucharest Early Intervention Project

**DOI:** 10.1016/j.dcn.2015.12.004

**Published:** 2015-12-13

**Authors:** Ross E. Vanderwert, Charles H. Zeanah, Nathan A. Fox, Charles A. Nelson

**Affiliations:** a School of Psychology, Cardiff University, 70 Park Place, Tower Building, Cardiff CF10 3AT, United Kingdom; b Tulane University, New Orleans, LA, United States; c University of Maryland, College Park, MD, United States; d Boston Children's Hospital and Harvard Medical School, Boston, MA, United States; e Harvard Graduate School of Education, Cambridge, MA, United States; f Harvard Center on the Developing Child, Cambridge, MA, United States

**Keywords:** Early deprivation, Foster care intervention, EEG, BEIP, Alpha activity, Beta activity

## Abstract

Extreme social and cognitive deprivation as a result of institutional care has profound effects on developmental outcomes across multiple domains for many abandoned or orphaned children. The Bucharest Early Intervention Project (BEIP) examines the outcomes for children originally placed in institutions who were assessed comprehensively and then randomized to foster care (FCG) or care as usual (CAUG) and followed longitudinally. Here we report on the brain electrical activity (electroencephalogram: EEG) of 12-year-old children enrolled in the BEIP. Previous reports suggested improvement in resting EEG activity for the group of children placed in the foster care intervention, particularly those placed before 24 months of age compared to children who were randomized to CAUG or those placed into families after this age. At 12 years, differences between those in the FCG and those in the CAUG persist in the alpha band (8–13 Hz), but not in higher frequency bands (i.e. in the beta band; 15–30 Hz), except in those children placed into the FCG who remained in high quality care environments over the course of the study. These findings highlight the importance of maintaining a stable high quality caregiving environment, particularly for children exposed to early psychosocial deprivation, for promoting healthy brain development.

## Highlights

• Reports EEG activity from 12-year wave of the Bucharest Early Intervention Project.
• Intervention effects in neural activity persist from 8-year wave to 12-year wave.
• Foster care children have greater alpha power compared to care-as-usual children.
• Stable foster care placement showed greater beta power versus disrupted placements.

## 1 Introduction

The use of institutional care for abandoned or orphaned infants and children has remained a common practice throughout the world (Browne et al., 2006, UNICEF, 2015); however, institutional environments are harmful for healthy physical and psychological development especially for young children. Many institutions have high child-to-caregiver ratios, have highly regimented schedules with extended periods where children are left alone, and very little engagement with the caregivers (Castle et al., 1999, Nelson, 2007, Smyke et al., 2007). The consequences of these rearing conditions often include physical growth restriction, a wide range of behavioral problems, and deficits in cognitive function compared to children raised in families (Maclean, 2003, Nelson, 2007; for review, see Nelson et al., 2014).

Extreme deprivation dramatically alters neural architecture and functioning. A number of neuroimaging studies have examined the impact of those early experiences in post-institutionalized adoptees. For example, structural magnetic resonance imaging (MRI) studies have shown decreased grey and white matter volumes (Mehta et al., 2009, Sheridan et al., 2012), increased amygdala volume (Mehta et al., 2009, Tottenham et al., 2010), decreased cerebellar volumes (Bauer et al., 2009), and disrupted connectivity between the frontal and temporal lobes (Eluvathingal et al., 2006) in previously institutionalized children compared to community controls. Together these studies demonstrate that typical neural development is altered as a result of early deprivation. However, fewer studies have examined the impact of intervention and tracked changes in brain development as a result of removal from institutions.

The BEIP is the first study to experimentally examine the physical, psychological, and neural sequelae of institutional rearing and the developmental trajectories of children removed and placed into a novel foster care intervention (Smyke et al., 2009, Zeanah et al., 2003). A group of infants living in institutions as well as an age-matched community control sample (never institutionalized group; NIG) all living in Bucharest, Romania were recruited into the study. After an initial screening to exclude for genetic disorders or other medical conditions the infants completed a baseline assessment, including resting EEG. The infants were then randomly assigned to either remain in the institution (care as usual; CAUG) or placed with a foster family (FCG). Random assignment of these children provides an opportunity to examine the effects of the intervention and repeated assessments over the children's life have allowed examination of changes in development associated with enrichment of their early care environment. Greater detail regarding the study design and ethical issues are described elsewhere (Miller, 2009, Millum and Emanuel, 2007, Zeanah et al., 2012).

Previous reports from this study found at the baseline assessment, infants living in institutions showed markedly decreased power in both alpha and beta activity and increased theta power compared to the never institutionalized community controls (Marshall, Fox and the BEIP core group, 2004). The pattern of higher theta and lower alpha power is one that is associated with attention-deficit/hyperactivity disorder (ADHD) and other learning disorders (Barry et al., 2003, Barry et al., 2009, Chabot et al., 2005, McLaughlin et al., 2010), the former being highly prevalent among previously institutionalized children (Bos et al., 2011, Kreppner et al., 2001, Wiik et al., 2011, Zeanah et al., 2009).

Following placement into foster care, EEG was again collected when the children were 42 months. The mean age of placement for the foster care group was 22 months. At the 42 month assessment there was a hint that earlier placement into foster care was beneficial, as infants placed at younger ages showed increasing alpha power relative to older placed infants (Marshall et al., 2008). The effect of timing of placement in foster care became clear by the time the children were 8-years-old; infants placed into foster care before 24 months were indistinguishable from children in the community while those placed after 24 months were identical to the CAUG (Vanderwert et al., 2010). Further, age of removal from institutions also significantly impacted the developmental trajectories of alpha and beta power between 42-months and 8-years (Stamoulis et al., 2015) suggesting ongoing plasticity in beta rhythm that was not detected in the follow-up at 8 years.

The aim of this study is to examine the continuing effects of foster care intervention on the neural activity of children removed from institutions in infancy. For the current study, resting EEG activity was acquired when children in the BEIP study were 12 years of age. Our primary hypothesis was that the intervention effects observed in the alpha band when the children were 8 years old would persist to the current assessment and that the intervention would also improve power in the beta band in the FCG compared to the CAUG. We also wanted to examine whether timing effects of the intervention (i.e., age at placement into foster care) would affect the pattern of alpha and beta activity.

We conducted two additional exploratory analyses. First, following up a recent examination of the developmental trajectories of the EEG from children in the BEIP study (Stamoulis et al., 2015), we also wanted to examine continuing changes in the three frequency bands between ages 8- and 12-years. Second, over the duration of the BEIP study some of the children in the FCG have experienced disruptions to their caregiving environment. Recently, Humphreys et al. (2015) found that children in stable placement in foster care had fewer internalizing and externalizing symptoms compared to those with unstable placements. Therefore, we also examined the effects of stability of placement on the EEG.

## 2 Methods

### 2.1 Sample

At the 12-year assessment, EEG was collected on 50 (26 male) children from the FCG, 49 (27 male) children in CAUG, and 48 (22 male) children from the NIG. The three groups did not differ on gender, *χ*^2^(2, *N* = 147) = .863, *p* = .65; or on age at data collection (*M* = 12.60, SD = .54; *F*(2, 146) = .324, *p* = .724). A number of children were excluded from analyses due to an excessive number of bad channels or too few artifact free epochs (>6 channels; *N* = 3 CAUG, 3 FCG, 2 NIG); the task not being completed due to equipment failure or subject non-compliance (*N* = 1 CAUG, 1 FCG); medication use (*N* = 7 CAUG, 5 FCG); or with EEG data exceeding ± 2 SD from the group mean in either absolute or relative power at multiple electrode clusters across the scalp, in multiple frequency bands, or meeting both these criteria (*N* = 5 CAUG, 3 FCG, 5 NIG). Of the original sample, 19 CAUG, 17 FCG, and 40 NIG children were unavailable for testing (Table 1).

**Table 1:** Continuity of participants from the 8-year to 12-year wave of assessments.

	Assessed at 8 yr & 12 yr	Assessed at 8 yr not 12 yr	Assessed at 12 yr not 8 yr	Missing at 8 yr & 12 yr	Mean (SD)
Group					Placement age
CAUG	42	6	7	13	–

FCG	45	8	5	9	22.62 (7.01)
Stable	21	0	3	2	21.50 (7.15)
Disrupted	24	2	1	1	23.81 (6.95)
NIG	29	13	19	27	–

*Note.* There are no differences between groups in the number of children assessed or missing at 8-year or 12-year waves. There are no differences in age of placement into the intervention between the FCG-Stable and FCG-Disrupted.

The University Institutional Review Boards of the principal investigators (Fox, Nelson, and Zeanah) and of the University of Bucharest approved the study protocol. Romanian law dictates that consent be given by the legal guardian of each child, therefore in the cases where consent was unavailable, assent was obtained from each caregiver who accompanied a child to the visit and from each child.

### 2.2 EEG recording & analysis

**Fig. 1:**
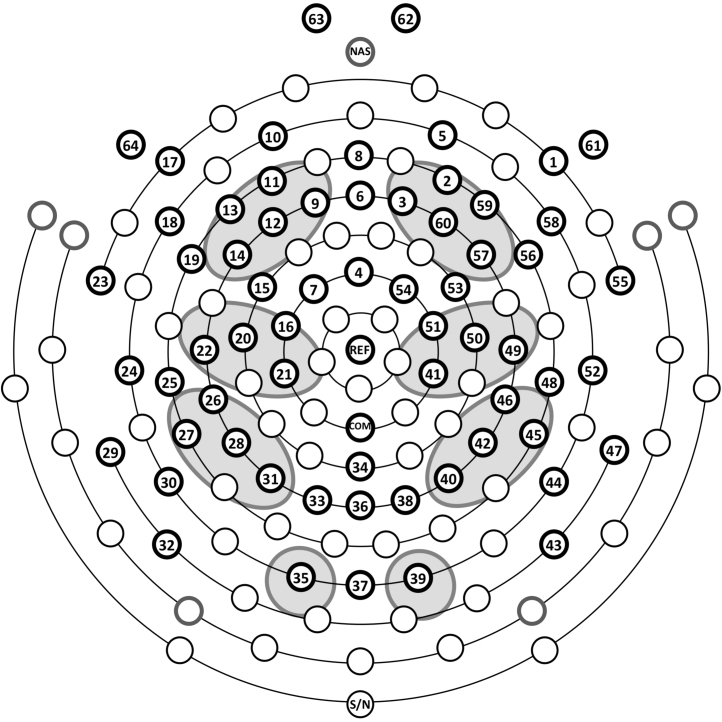
Example of the 64-channel EEG net with clusters highlighted in grey. Frontal clusters are centered on channels 12 and 60; central on 20 and 50; parietal on 28 and 42; and occipital on channels 35 and 39.

Continuous EEG was recorded using a 64-channel HydroCel Geodesic Sensor Net (Electrical Geodesic Inc., Eugene, OR) that was connected to a NetAmps 300 amplifier (Electrical Geodesic Inc., Eugene, OR) and referenced online to a single vertex electrode (Cz). Channel impedances were kept at or below 50 kΩ and signals were sampled at 500 Hz. The child's head circumference was measured and an appropriately sized net was fitted. EEG was recorded while the children sat quietly in a chair, alternating 1-min epochs of eyes open and eyes closed for a total of 6 min. During the eyes open condition, children were instructed to fixate on a small white cross on the center of a computer screen. EEG data were preprocessed offline using NetStation 4.5 (Electrical Geodesic Inc., Eugene, OR). The EEG data were first bandpass filtered (.3 to 100 Hz), bad channels were then identified and replaced, and an average reference was computed. The EEG signals were then exported to Matlab for artifact rejection and power analysis. Blinks and other artifacts were automatically identified with a threshold of ±100 μV and excluded from analyses. The clean data were then submitted to a Fast Fourier transform (FFT) with a 1-s Hanning window and 50% overlap. The CAUG (*M* = 269.12, SD = 46.75), FCG (*M* = 259.39, SD = 64.80), and NIG (*M* = 282.44, SD = 48.17) contributed an equal number of windows for analysis (*F*(2, 111) = 1.81, *p* = .17). Spectral power (μV^2^) in the eyes open condition was computed for the following bands: theta (4–7 Hz), alpha (8–13 Hz), and beta (15–30 Hz). The eyes open condition was chosen for consistency with previous assessments (Marshall and Fox, 2004, Marshall et al., 2008, Vanderwert et al., 2010). The power in each frequency band was then averaged over clusters of channels approximating the international 10–20 locations F3, F4, C3, C4, P3, P4, O1, and O2 (Fig. 1).

For each cluster and band, we examined both absolute power (the natural logarithm of the spectral power) and relative power (the proportion of power in each band relative to the total power between 4 and 30 Hz). Developmentally, there is a shift from dominance in lower frequency bands (e.g. greater delta and theta power) to higher frequencies (i.e. alpha power); relative power is an ideal index to measure these developmental changes while minimizing inter-individual differences in absolute power due to maturational factors, such as skull thickness (Somsen et al., 1997). However, because relative power is a proportion score, changes in absolute power in one band affects relative power values in the other bands, therefore, absolute power is helpful in identifying the specific band(s) that result in changes in relative power. We present the results for both absolute and relative power following the procedures at previous assessments (Marshall and Fox, 2004, Marshall et al., 2008, Vanderwert et al., 2010).

### 2.3 Analysis approach

**Fig. 2:**
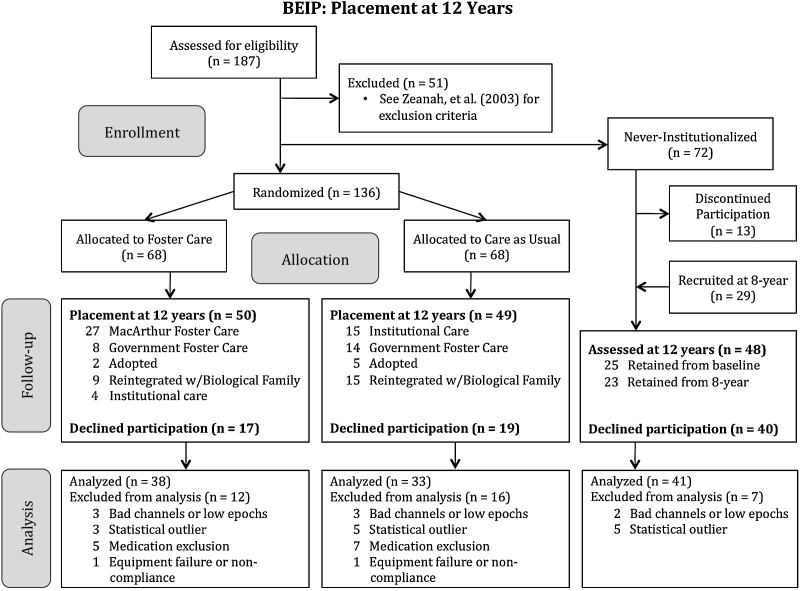
Group status at 12-years for the care as usual, foster care, and never-institutionalized groups in the BEIP study. Three children in foster care were moved from one placement to another and were therefore placed in the disrupted foster-care group.

In order to test our hypothesis that there was an intervention effect on the EEG, our first set of analyses employ an intent-to-treat approach based on the design of the BEIP project as a randomized, controlled trial of foster care as an intervention for institutionalized children. In the intervening years since the outset of the study, a number of cultural and political changes in Romania have resulted in the formation of a national foster care program with the aim of decreasing the number of infants and children living in institutions. As a result of these policies, many of the CAUG and FCG children have had changes in their living situations; being removed from institutions or their study foster families and placed in government foster care homes or reunited with their biological families (see Fig. 2).

Intent-to-treat analysis treats each child's data as if they had remained within their randomly assigned groups. These effects are, therefore, a conservative estimate of the intervention effect, and we considered *α* < .10 of interest for follow-up examination. Differences in EEG power were examined with separate repeated measures analyses of variance (RM-ANOVAs) for each band with 4 regions (frontal, central, parietal, occipital) and 2 hemispheres (left, right) as within-subjects factors and placement group (CAUG, FCG) as the between subjects factor. Greenhouse–Geisser correction was used for violations of sphericity. Independent samples *t*-tests were used for any post hoc comparisons. Note that interactions not involving group are not reported. For those frequency bands where an intervention effect was found we then examined the scalp activity of the intervention groups as they compared to the never-institutionalized children.

We next considered age-at-placement effects in the FCG on the EEG measures using correlation analyses to identify any frequency bands that were sensitive to the age the child was removed from the institution.

A subsample of children provided EEG data from both when they were 8-years-old and at the current assessment. EEG was collected using identical procedures at both assessments (Vanderwert et al., 2010), however, there was a change in the EEG acquisition systems. Therefore, to examine changes in the EEG between 8 and 12 years, we used the relative power metric, which better controls for acquisition system, analysis software, and frequency band differences.

A recent study found that children who remained in stable foster care had improved mental health outcomes compared to those who had multiple disruptions in their care (Humphreys et al., 2015). These disruptions in care may impact neural development; therefore, we also examined the effects of placement stability in each of the three frequency bands. Just over half of the children in the FCG remained with their original foster parent at age 12 years (see Table 1). Therefore, we set aside the original intent-to-treat groupings and examined the effects of the stability foster care placements for children in the FCG group only, separating the FCG into those children who remained in their original BEIP foster placement (FCG-Stable) and those who experienced disruptions in their placements (FCG-Disrupted; Humphreys et al., 2015, Rutter, 2015).

## 3 Results

### 3.1 Intent-to-treat analyses

For the intent-to-treat analyses, each band was analyzed separately with a 4 region (frontal, central, parietal, occipital) × 2 hemisphere (left, right) × 2 group (CAUG, FCG) RM-ANOVA.

#### 3.1.1 Absolute power

Examination of absolute power revealed no main effects or interactions involving the intent-to-treat groupings in any of the three bands.

#### 3.1.2 Relative power

*Theta band.* Examination of intervention effects in relative theta power revealed a marginally significant main effect of group (*F*(1, 69) = 3.32, *p* = .073, *η*^2^_*p*_ = .063). Examination of the group means revealed that the CAUG (*M* = .41, SD = .09) had greater power compared to the FCG (*M* = .37, SD = .09).

To examine the intervention effect of relative theta power within the context of the never-institutionalized group we computed average power across the scalp and employed an univariate ANOVA with the averaged relative theta power as the dependent variable and the three groups (CAUG, FCG, and NIG) as the between subjects variable. Results of the analysis revealed a significant main effect of group (*F*(3, 109) = 741.96, *p* < .001, *η*^2^_*p*_ = .953). Post hoc examination of the main effect revealed that the CAUG had greater power compared to the FCG (*t*(69) = 1.95, *p* = .055) and the NIG (*M* = .35, SD = .07; *t*(72) = 2.60, *p* = .011). There was no difference in power between the FCG and NIG (*t*(77) = .63, n.s.).

*Alpha band.* Examination of relative alpha power revealed a marginally significant main effect of group (*F*(1, 69) = 3.88, *p* = .053, *η*^2^_*p*_ = .053). Examination of the group means revealed that the CAUG (*M* = .37, SD = .10) had less power compared to the FCG (*M* = .42, SD = .09).

**Fig. 3:**
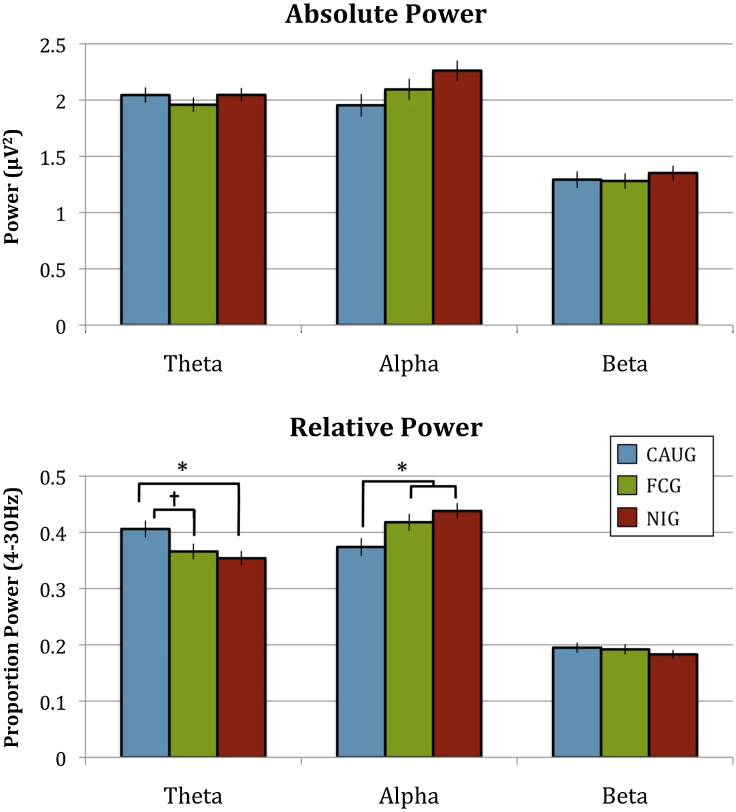
Mean absolute (top) and relative (bottom) power from the theta (4–7 Hz), alpha (8–13 Hz), and beta (15–30 Hz) bands for the care-as-usual group (CAUG), foster care intervention (FCG), and never-institutionalized community controls (NIG). * *p* < .05, † *p* < .10.

To examine the intervention effect of relative alpha power within the context of the never-institutionalized group we computed average power across the scalp and employed an univariate ANOVA with the averaged relative alpha power as the dependent variable and the three groups (CAUG, FCG, and NIG) as the between subjects variable. Results of the analysis revealed a significant main effect of group (*F*(3, 109) = 789.04, *p* < .001, *η*^2^_*p*_ = .956). Post hoc examination of the main effect revealed that the CAUG had less power compared to the FCG (*t*(69) = 2.10, *p* = .040) and the NIG (*M* = .44, *SD* = .08; *t*(72) = 3.05, *p* = .003). There was no difference in power between the FCG and NIG (*t*(77) = 1.00, n.s.; Fig. 3).

*Beta band.* Examination of intervention effects in relative beta power found no main effects or significant interactions with group.

### 3.2 Age-at-placement

To examine possible sensitive periods in cortical development, we computed correlations between the average absolute and relative power in each frequency band and the age at placement for the FCG. No significant associations were detected.

### 3.3 Continuity of EEG power

**Fig. 4:**
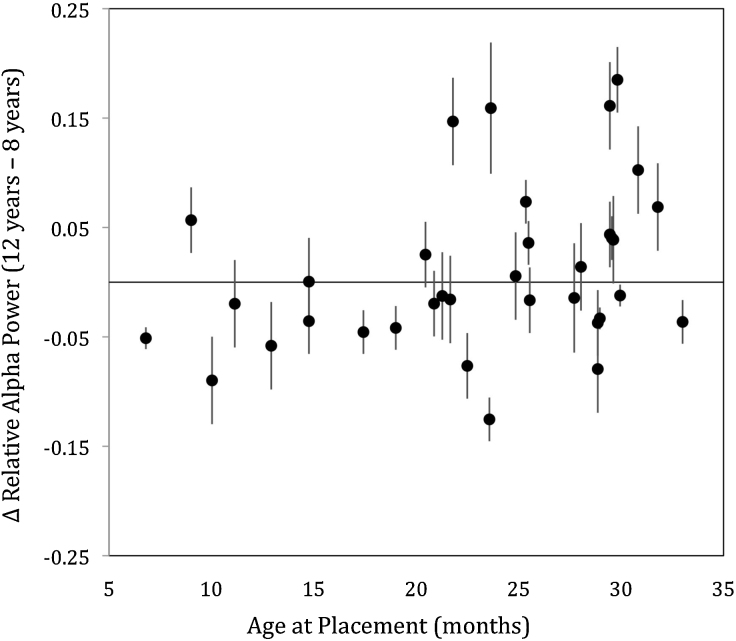
Relation between change in relative alpha power and age at placement into foster care for the FCG. Grey bars are the pooled standard error across the scalp across time points.

Continuity of relative power in each band was examined using Pearson product-moment correlations within each group between relative power at 8 years and relative power at 12 years in each band. Relative power in each band was significantly correlated between the two time points in both the CAUG and FCG groups in all three bands. Relations between 8-year and 12-year assessments for the NIG were limited to the alpha and beta bands (Table 2). To examine whether the change in relative power was related to age at placement into foster care, we calculated the difference in relative power (12–8 years) for each band and ran additional correlations with Age-at-Placement. The change in relative power in the alpha band was the only significant correlation with the FCG Age-at-Placement (*r*(35) = .333, *p* = .051; Fig. 4).

**Table 2:** Correlations between relative power at 8 year and 12 year assessments.

Group	*n*	Theta	Alpha	Beta
CAUG	29	.728***	.796***	.504**
FCG	35	.732***	.616***	.769***
Age at placement		−.272	.333*	−.182
NIG	24	.367	.562**	.500*

*Note.* Correlations reported for age at placement in the FCG are based on the change in relative power from 8 to 12 years.

*** *p* < .001.

** *p* < .01.

* *p* = .051.

#### 3.3.1 Group differences in changes in relative power

We next analyzed group differences in relative power change between 8- and 12-years. Because there were no group differences in region or hemisphere at either assessment time point, we averaged the relative power across the scalp to examine scalp-wide changes in band power. We employed a 2 Time (8-year, 12-year) × 3 Band (Theta, Alpha, Beta) × 3 Group (CAUG, FCG, NIG) RM-ANOVA.

The analysis revealed significant main effects of Time (*F*(1, 85) = 607.75, *p* < .001, *η*^2^_*p*_ = .88) and Band (*F*(2, 170) = 208.84, *p* < .001, *η*^2^_*p*_ = .71) qualified by Band × Time (*F*(2, 170) = 20.50, *p* < .001, *η*^2^_*p*_ = .194) and Band × Group (*F*(4, 170) = 5.27, *p* = .001, *η*^2^_*p*_ = .110) interactions. Post hoc analyses of the Band x Time interaction revealed a significant decrease in power between Time 1 (*M* = .42, SD = .08) and Time 2 (*M* = .37, SD = .09; *t*(85) = 7.04, *p* < .001) in the theta band and a significant increase between Time 1 (*M* = .17, SD = .05) and Time 2 (*M* = .19, SD = .05; *t*(85) = 3.05, *p* = .003) in the beta band. There were no significant changes in the alpha band.

Post hoc analyses for the Band x Group interaction revealed that in the theta band, the CAUG (*M* = .43, SD = .08) had higher relative power compared to the FCG (*M* = .39, SD = .08; *t*(62) = 1.89, *p* = .062) and the NIG (*M* = .36, SD = .07; *t*(51) = 3.30, *p* = .002) and no difference between the FCG and NIG (*t*(57) = 1.50, *p* = .14). In the alpha band, the CAUG (*M* = .37, SD = .08) had significantly lower relative power compared to the FCG (*M* = .42, SD = .08; *t*(62) = 2.47, *p* = .017) and the NIG (*M* = .45, SD = .08; *t*(51) = 3.71, *p* < .001) and no difference between the FCG and NIG (*t*(57) = 1.55, *p* = .127). There were no differences between the groups in the beta band. Plots of the individual-level changes in relative power in each band are available in Supplementary materials.

### 3.4 Effects of placement stability

To examine placement stability, each band was analyzed separately with 4 region (frontal, central, parietal, occipital) × 2 hemisphere (left, right) × 4 placement stability (CAUG, FCG-Disrupted, FCG-Stable, NIG) RM-ANOVAs.

#### 3.4.1 Absolute power

*Theta band.* There were no main effects or significant interactions with placement stability in absolute theta power.

*Alpha band.* There were no main effects or significant interactions with placement stability in absolute alpha power.

*Beta band*. Examination of absolute beta power revealed a main effect of placement stability (*F*(3, 107) = 4.23, *p* = .028, *η*^2^_*p*_ = .081) qualified by a hemisphere × stability interaction (*F*(3, 107) = 3.52, *p* = .018, *η*^2^_*p*_ = .090). Follow-up analyses of the main effect revealed the FCG-Disrupted (*M* = 1.10, SD = .41) had significantly less beta power than the FCG-Stable (*M* = 1.50, SD = .41; *t*(35) = 2.97, *p* = .005) and NIG (*M* = 1.35, SD = .41; *t*(59) = 2.27, *p* = .027). There were trend differences between the CAUG (*M* = 1.29, SD = .41) and both the FCG-Disrupted (*t*(51) = 1.68, *p* = .099) and the FCG-Stable (*t*(48) = 1.69, *p* = .097). All other contrasts were not significant.

Post hoc analyses of the interaction revealed that the FCG-Stable had significantly greater beta power in both hemispheres compared to the FCG-Disrupted (Left: *t*(35) = 3.61, *p* < .001; and Right: *t*(35) = 2.08, *p* = .045); the FCG-Disrupted had significantly less beta power in the left hemisphere and marginally less power in the right compared to the NIG (Left: *t*(59) = 2.45, *p* = .017; and Right: *t*(59) = 1.93, *p* = .058); and in the left hemisphere, the CAUG had significantly more power than the FCG-Disrupted (*t*(51) = 2.08, *p* < .042) and significantly less power than the FCG-Stable (*t*(48) = 2.02, *p* = .049). All other contrasts were not significant.

#### 3.4.2 Relative power

*Theta band.* Examination of relative theta power revealed a significant main effect of placement stability (*F*(3, 107) = 2.77, *p* = .045, *η*^2^_*p*_ = .072). Post hoc t-tests revealed the CAUG had greater relative power compared to the FCG-Stable and the NIG (*t*(48) = 2.16, *p* = .036 and *t*(72) = 2.43, *p* = .017, respectively). All other contrasts were not significant.

*Alpha band.* There were no main effects or significant interactions with placement stability in relative alpha power.

**Fig. 5:**
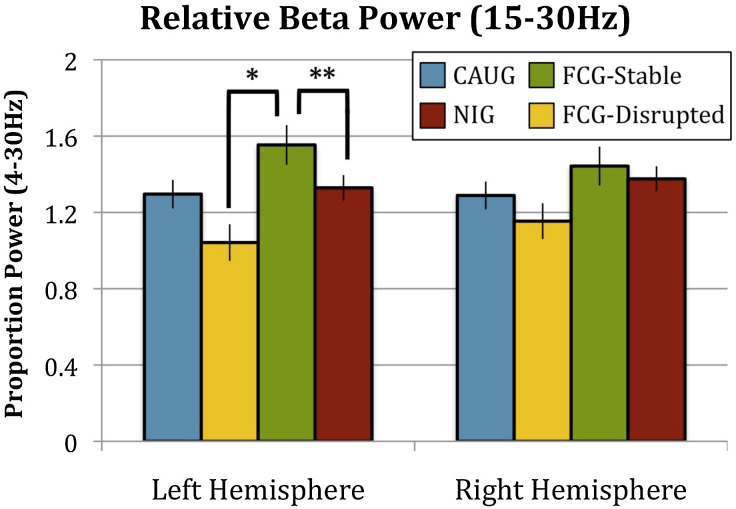
Relative beta power in the left and right hemispheres between placement stability groups. * *p* < .05; ** *p* < .01.

*Beta band*. Examination of relative beta power revealed a significant hemisphere by placement stability interaction (*F*(3, 107) = 3.80, *p* = .012, *η*^2^_*p*_ = .096; Fig. 5). Post hoc t-tests revealed the FCG-Stable group had significantly greater relative power in the left hemisphere compared to the FCG-Disrupted and NIG (*t*(35) = 2.61, *p* = .013 and *t*(56) = 2.73, *p* = .008, respectively). All other contrasts were not significant.

## 4 Discussion

The aim of this study was to examine the continuing impact of a foster care intervention on neural activity following early psychosocial deprivation. At 12 years, children who were removed from institutions and placed into foster care demonstrated continuing benefits as a result of intervention as indexed by their resting EEG activity; specifically, children placed into high quality foster care displayed marginally decreased theta power and elevated alpha power compared to children who received care as usual. This finding is illustrated by the measures of relative power where the FCG children resembled the NIG children with an increasing proportion of power in the alpha band while the CAUG showed an immature pattern of brain activity with greater distribution of power in the theta band (Benninger et al., 1984, Matthis et al., 1980, Raine et al., 2001).

The distribution of greater power in slower frequencies of the EEG is not only characteristic of extreme deprivation or social isolation (Gendreau et al., 1972, Marshall and Fox, 2004, Zubek et al., 1963), but has been observed in typically developing populations of children living in impoverished environments. A number of studies have shown that children living in low resource homes (e.g., poor nutrition, unsanitary living conditions, less responsive or sensitive caregiving) show a similar “immature” pattern of EEG activity as we have observed in the BEIP sample. For example, Otero and colleagues (Otero, 1994, Otero, 1997, Otero et al., 2003), have followed a sample of middle-low and low socioeconomic status infants from 18-months to 6 years and measured EEG at three different time points and found that children with lower resources – compared to children with more cognitive and social stimulation through parental involvement – demonstrated greater EEG activity in slower frequencies (delta and theta bands) and lower power in higher frequencies often associated with better attention and cognitive performance (alpha and beta bands; Fernandez et al., 2000). Indeed, an enriched nursery school intervention has been shown to alter the neural activity of impoverished children, increasing power in fast EEG activity and improved attentional orienting during a cognitive task (Raine et al., 2001); suggesting that early intervention may improve outcomes for all children.

There appears to be a high degree of stability in the EEG trajectories between the 8- and 12-year assessments. A recent study examined the developmental trajectories of EEG activity in the BEIP sample from baseline through 8 years (Stamoulis et al., 2015). They found that intervention group assignment was significantly related to the trajectories of the alpha and beta bands. There are differences in the methods that preclude direct comparison, however, examination of the changes in the EEG between 8 and 12 years suggest that all groups continue to show maturational changes in the EEG that remain dependent upon early experiences consistent with those reported by Stamoulis and colleagues. Moreover, the decreases in relative theta, increases in relative beta, and stability of relative alpha power within this age range are consistent with other longitudinal studies of EEG in typically developing children (Benninger et al., 1984, Clarke et al., 2001, Matthis et al., 1980).

The results of the current 12-year follow up study demonstrate continuity of intervention effects that were found at 8 years. When children were observed at age 8 those placed into foster care before 24 months were indistinguishable to never institutionalized children in alpha power (Vanderwert et al., 2010). The lack of a significant difference between the FCG and NIG in either absolute or relative power in any of the frequency bands and no relations between EEG and the age at placement into foster care suggests that continued high quality care likely improves the neurodevelopmental outcomes of children who have experienced early social and cognitive deprivation.

There is one caveat to this interpretation. When we set aside intent-to-treat and examined the relations between EEG and stability of care versus any foster care experience, the results suggest that maintaining a stable caregiving environment had a greater impact on EEG activity than for children who experienced disruptions in caregiving. When we split the FCG into those who remained with their initial caregivers and those who changed caregivers, through, for example, adoption or reintegration with their biological family, our predicted improvements in beta activity were observed only in those children who remained in stable, high-quality care. These improvements in brain activity mirror recent evidence that stability of care decreased internalizing and externalizing symptoms in this sample at 12 years (Humphreys et al., 2015).

The heightened beta band activity for the FCG-Stable children is encouraging, as beta activity has been associated with better performance on tasks that require increased cognitive load such as verbal and spatial working memory (Fernandez et al., 2000), motor performance (Pfurtscheller et al., 1996), language processing (Papanicolaou et al., 1986), and processing emotionally valenced stimuli, including faces or IAPS stimuli (Guntekin and Basar, 2014, Ray and Cole, 1985). A recent review on the function of beta activity suggests its role in cognitive control and maintenance of a current behavioral state (Engel and Fries, 2010). Thus, increased resting beta band activity may reflect compensatory top-down control mechanisms required to follow the experimenter's instructions to sit still and remain quiet during the testing session. Alternatively, the greater power in the beta band in the FCG-Stable children may be indicative of *functional potential* for improvements in other domains.

The neural mechanisms affected by the intervention are unclear; however, a recent structural MRI study of these same BEIP children identified a plausible anatomical explanation. Sheridan et al. (2012) found that white matter volume mediated the BEIP intervention effect on alpha power in children at 8 years of age. They concluded that institutional care may have resulted in the delay of white matter maturation and earlier intervention may prevent loss of plasticity. One possibility for this finding is that age of intervention may not affect plasticity per se but affect the rate at which white matter matures.

An alternative interpretation, in light of the placement stability findings, is that stable, high-quality caregiving may support continued white matter development. There is very little evidence in support of this interpretation as only a handful of studies have examined caregiving and cortical development. In a structural MRI study of 10-year-old children, the family's income-to-needs ratio predicted both white and gray matter volume (Luby et al., 2013). Income-to-needs is a proxy for the cluster of experiences associated with poverty; among these are stressed caregiving and multiple disruptions to housing and caregivers, however, interpretation of these data should be approached with caution as there are still a number of concomitant factors (e.g. educational resources, nutrition, etc) that likely also contribute to these findings. More directly relating caregiving to cortical development, an intervention study training sensitive and responsive caregiving behaviors in parents of preterm infants found increased white matter volume, compared to the control infants, within the first few months of the intervention (Als et al., 2004). While this demonstrates remarkable plasticity in cortical development, this intervention focused on the first few months of life, likely prior to the closure of any sensitive period for white matter development. The finding of stable foster care enhancing brain activity adds to other research documenting the importance of stability for improving emotion regulation, stress reactivity, inhibitory control, and internalizing and externalizing symptomology (Blair and Raver, 2012, Dozier et al., 2006, Lewis et al., 2007, Humphreys et al., 2015) and will be an important direction for future studies of neural development.

One limitation of this study is the use of resting EEG as an index of neural activity. Without specific tasks it is difficult to derive any conclusions regarding the functional benefits of the improved EEG power as a result of the intervention. For example, Eluvathingal et al. (2006) have identified increased cortico-cortical connectivity in previously institutionalized children that may be structurally compensatory but provide no improved cognitive functioning. It is possible that the differences we observed at age 12 may be a result of similar compensatory circuits, which could account for the improved EEG activity but may not impart any social or cognitive advantages.

The data from this current study suggest that a family based foster care intervention administered up through when children were 54 months of age had continued effects on EEG activity some 8 years later. At 12 years, the children in the foster care group show decreased theta power and increased alpha power that is comparable to never-institutionalized children compared to institutionalized children who did not receive the intervention. The expected intervention effects in the beta band were not observed outright, however, when we broke intent-to-treat, children in the intervention group who remained in their original placement had significantly greater beta band power, compared to those children that changed caregivers or did not receive the intervention. These improvements in neural activity may be important for the children's continuing development as they enter puberty or schooling transitions.
