# Corrigendum to "Normalization of EEG activity among previously institutionalized children placed into foster care: A 12-year follow-up of the Bucharest Early Intervention Project” [Dev. Cognit. Neurosci. 17 (2016) 68–75]

**DOI:** 10.1016/j.dcn.2024.101361

**Published:** 2024-03-06

**Authors:** Ross E. Vanderwert, Charles H. Zeanah, Nathan A. Fox, Charles A. Nelson

**Affiliations:** a School of Psychology, Cardiff University, 70 Park Place, Tower Building, Cardiff CF10 3AT, United Kingdom; b Tulane University, New Orleans, LA, United States; c University of Maryland, College Park, MD, United States; d Boston Children's Hospital and Harvard Medical School, Boston, MA, United States; e Harvard Graduate School of Education, Cambridge, MA, United States; f Harvard Center on the Developing Child, Cambridge, MA, United States

The authors regret that the y-axis should have gone from 0 to 0.25. The correct version of the Fig. 5 is shown below. The data analyses were correct as were the conclusions of the paper.

**Fig. 5:**
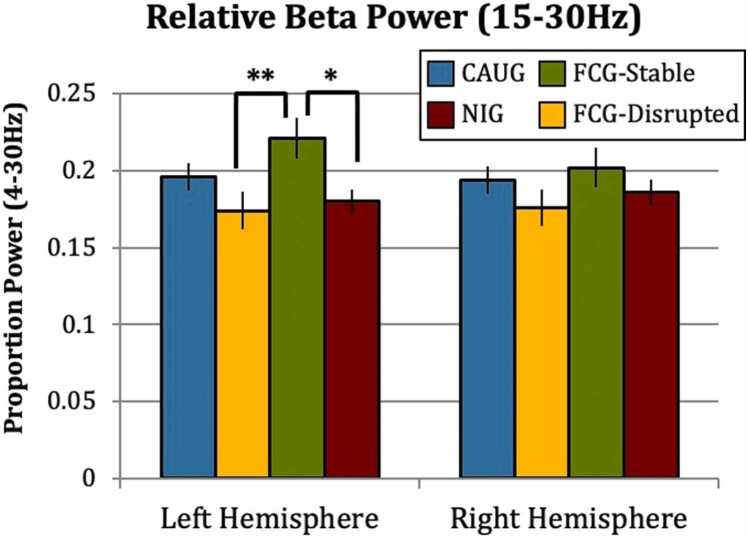
Relative beta power in the left and right hemispheres between Placement Stability groups. * *p* <.05; ** *p* <.01. The authors would like to apologise for any inconvenience caused.

