# Supplementary material for: Normalization of EEG activity among previously institutionalized children placed into foster care: A 12-year follow-up of the Bucharest Early Intervention Project
Source: Dev Cogn Neurosci. 2015 Dec 13;17:68–75. doi: 10.1016/j.dcn.2015.12.004 (PMC4727988; doi:10.1016/j.dcn.2015.12.004)
Supplement: Supplementary file 1 [file mmc1.doc]

Supplementary Materials


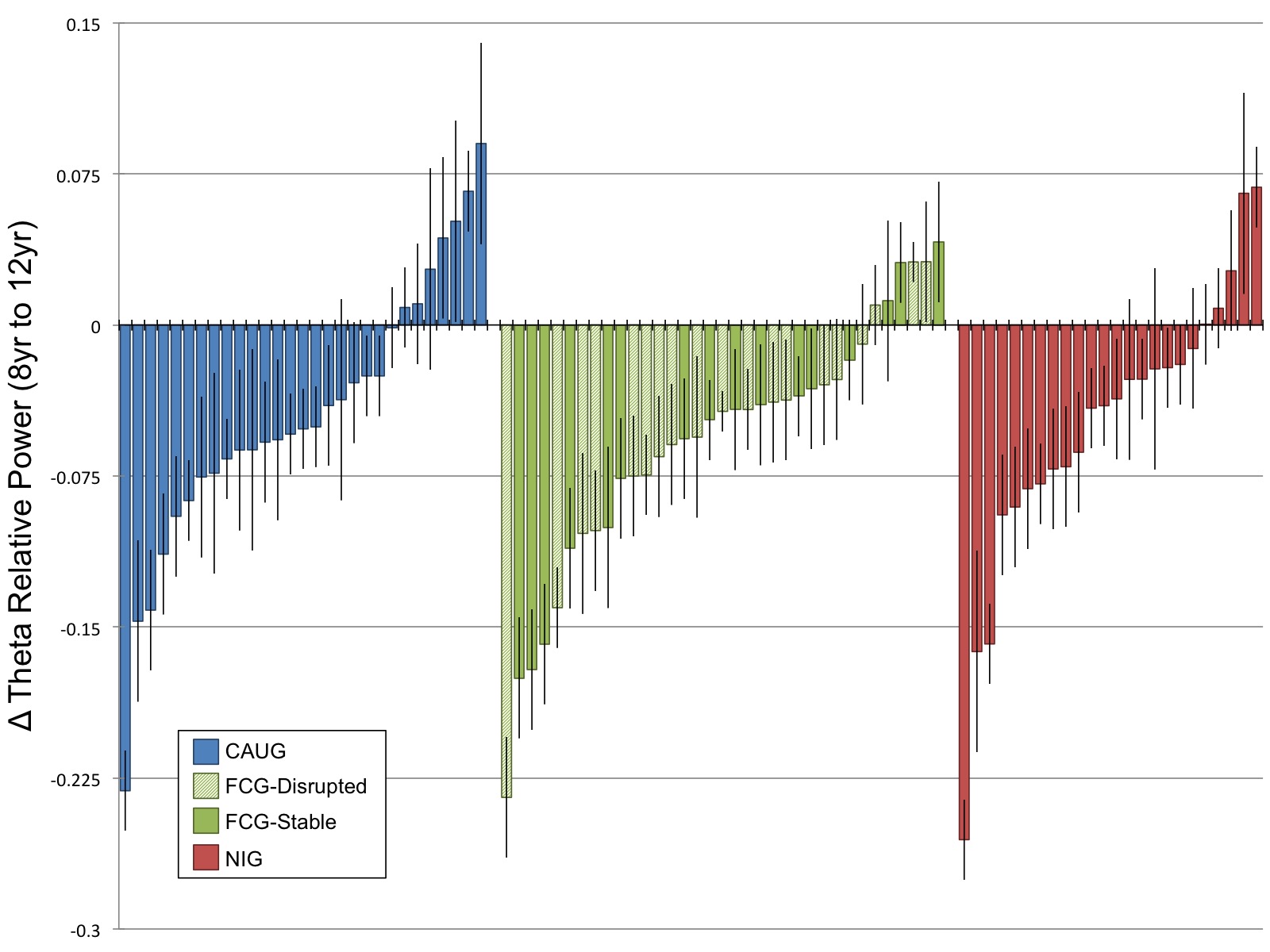
SM Figure 1. Change in relative Theta power in each individual between 8- and 12-years (12year – 8year). Standard error bars represent variability across the scalp between the two time points. Blue bars are the care as usual group (CAUG); green bars are for the foster care group (FCG), solid bars for FCG-Stable and stripped bars for FCG-Disrupted; and Red bars are the never institutionalized group (NIG).


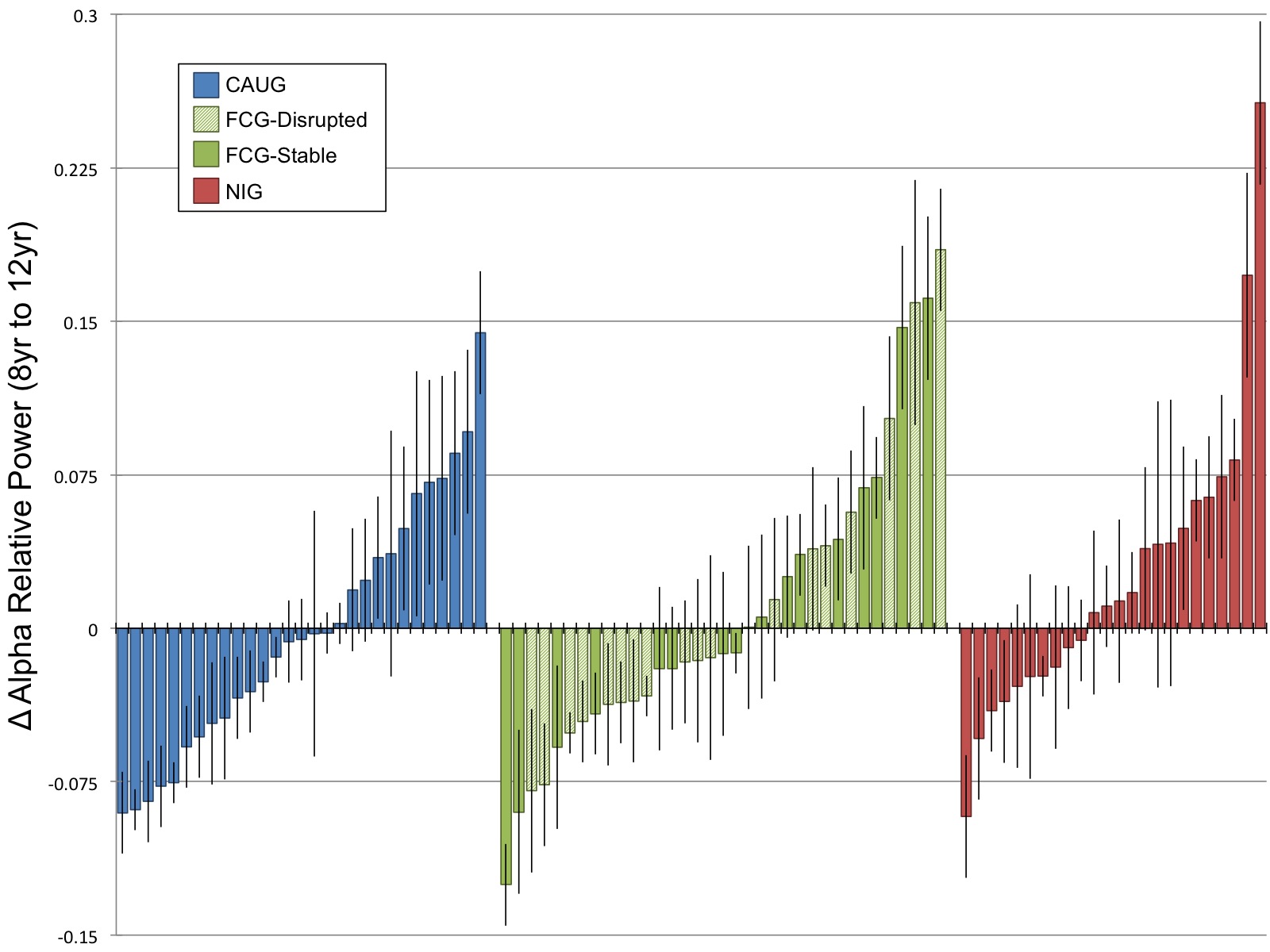
SM Figure 2. Change in relative Alpha power in each individual between 8- and 12-years (12year – 8year). Standard error bars represent variability across the scalp between the two time points. Blue bars are the care as usual group (CAUG); green bars are for the foster care group (FCG), solid bars for FCG-Stable and stripped bars for FCG-Disrupted; and Red bars are the never institutionalized group (NIG).


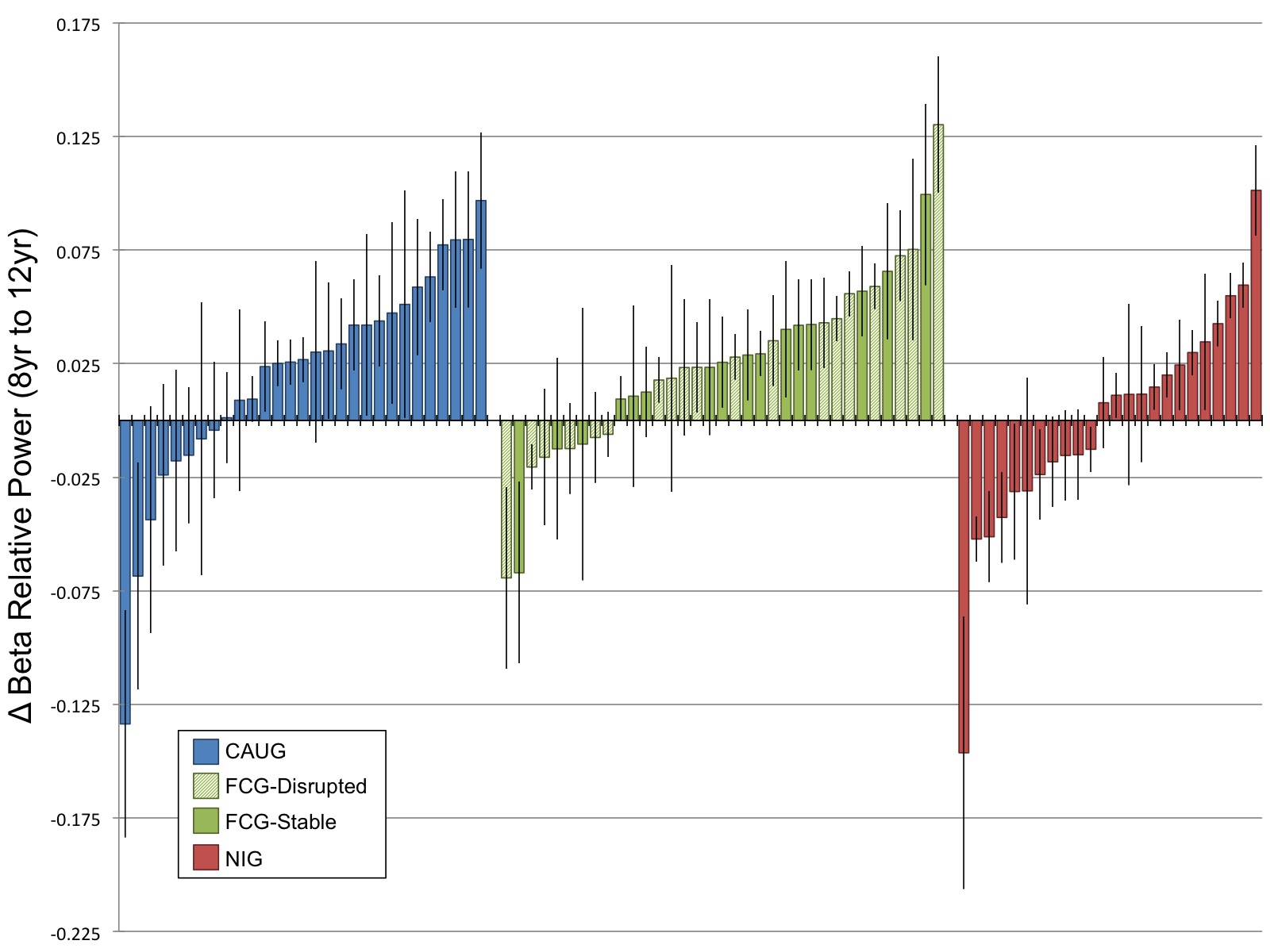
SM Figure 3. Change in relative Beta power in each individual between 8- and 12-years (12year – 8year). Standard error bars represent variability across the scalp between the two time points. Blue bars are the care as usual group (CAUG); green bars are for the foster care group (FCG), solid bars for FCG-Stable and stripped bars for FCG-Disrupted; and Red bars are the never institutionalized group (NIG).
